# Immigrants and health system challenges to TB control in Oman

**DOI:** 10.1186/1472-6963-10-210

**Published:** 2010-07-16

**Authors:** Abdullah Al-Maniri, Grethe Fochsen, Omar Al-Rawas, Ayesha De Costa

**Affiliations:** 1 College of Medicine and Health Sciences, Sultan Qaboos University, Muscat, Sultanate of Oman; 2 Department of Public Health Sciences, Division of Global Health, Karolinska Institutet, Stockholm, Sweden; 3 SIDA, Stockholm, Sweden

## Abstract

**Background:**

During the past three decades, Oman has made significant progress in controlling TB within it's borders. However, the national TB control program elimination target has yet to be reached. This study aims to explore the perceived roles played by the immigrant population and the private health sector in relation to TB control in Oman.

**Methods:**

We conducted seventeen interviews with different health care providers. The verbatim transcripts were processed using content analysis.

**Results:**

Three main themes emerged. Firstly the threat of repatriation faced by underprivileged expatriates, secondly the criticized and forgotten private health sector as a key player and thirdly the user and provider barriers faced by Omani patients in the Omani public health system.

**Conclusions:**

The study has identified some of the challenges and barriers to TB control in Oman. These challenges are mainly related to unintended negative consequences arising from the current repatriation policy of immigrants and to and the lack of involvement of the private sector in TB control. TB control strategies designed to address these challenges are needed, for Oman to reach its TB elimination targets.

## Background

The Sultanate of Oman, a high middle-income country, is located in the southeastern corner of the Arabian Peninsula. Oman has experienced dramatic economic change over the last 40 years, due in large part to its oil revenues, which were its main source of income. In 1970, the Gross Domestic Product (GDP) per capita was 158 Omani Rial (OR) (1 OR ≈ 2.7 US $). In 2006 the GDP per capita rose to 5319 OR. This economic change has been accompanied by major changes in key health indicators. For instance, the infant mortality rate has declined from 159 per 1000 live births in 1970 to 10.2 per 1000 live births in 2006[1]. In addition, Oman has made significant achievements with regard to the control of infectious diseases [2].

In 2007, the total population of Oman was estimated to be approximately 2.7 million of which 30% were non-Omani (expatriates). The Muscat region is considered to be the political and administrative hub of Oman. Around 44% of all expatriates in Oman live in Muscat, and most of these are labor workers. More than 44% of all private health care establishments in the country are in Muscat [3].

In Oman, health care is provided by public and private health systems. The public health care system is normally utilized by all nationals and immigrants, (commonly known in Oman as expatriates or guest workers), working in the government sector. Meanwhile, private health care provision in Oman has been increasing over the last 10 years. These services are utilized by many nationals and expatriates working in the private sector [4].

Primary public health care providers, mainly general practitioners, are responsible for pulmonary TB detection and diagnosis. Hospitals at the secondary and tertiary levels are primarily providing Directly Observed Treatment Short course (DOTS) for the first 2 months. The last 4 months of the DOTS are carried out in primary health care public sector settings. Identified or suspected TB cases diagnosed by the private health care system have to be immediately referred to the public health system for treatment and follow up. Private pharmacies are not allowed to purchase or sell any TB medication. All TB notifications and TB related policies and activities are regulated centrally by the National TB Control Program (TBCP) which was established in 1981[5]. The public health system provides free TB treatment to everyone [5].

Oman has been able to reduce the burden of TB by 85% in less than 25 years [6]. However, the TB elimination target (of 3 smear positive cases per 100,000 people by the year 2005 set in 1996 by the national TB control program), has not yet been reached, and concerns have been mounting. This is why recommendations have been made to the TB Control Program (TBCP) to stengthen TB control and to explore the obstacles to TB elimination [7].

Migration and immigrants are known to pose many challenges to TB control and elimination programs in low incidence countries [8] such as Oman. Around one third of Omani inhabitants are immigrants from the Indian subcontinent [3], an area which has a high prevalence of TB. Low incidence countries have different policies with regard to TB control among immigrants [9]. In Oman, expatriate patients who are identified as having TB are deported from the country after initiation of treatment and conversion to smear negative. The impact of this policy and the role of expatriates who are mainly served by an expanding private health care sector in relation to TB control have not been previously explored in Oman. This study explores health care provider's (private and public) perceptions and experiences with regard to health system and patient barriers to TB control in Oman. The study also explores the perceived roles of expatriates and of the private health sector in TB control in the Omani context.

## Methods

### Participants and data collection

The target groups of this study were health care providers (both public and private) who encounter TB cases during diagnosis and/or treatment. We purposively selected and interviewed 17 health care providers. The first author made a list of potential participants with support from colleagues in the Ministry of Health and in private practices. Potential participants were selected to reflect the public and private sectors, as well as different professional backgrounds; General Practitioners (GPs), physicians and nurses. All potential participants were required to have experience of working with tuberculosis for at least five years, interest and knowledge in the issues under investigation and consented to participate. The GPs and the nurses were TB control focal points in the health centers. The physicians were specialists and consultants in infectious diseases or pulmonary medicine. The total number of participants was based on reaching data saturation.

The first author contacted, communicated telephonically with potential participants to set appropriate dates and times for the interviews. Four of the interviewees were senior specialists and consultants and another four were public General Practitioners. In addition, five private GPs were included in the study. We also interviewed four public TB focal point nurses. Three of the public senior specialists and all the private practitioners were men. All the nurses were women. Two of the GPs were women and the other two were men. Two public GPs, four private GP's and one nurse were non-Omani. Potential participants were initially contacted by telephone. Those who agreed to participate were then interviewed individually at a time and place of their own choosing. Consent was obtained from all the participants, who were informed of the objectives and duration of the study. They were also informed that the interviews would be digitally recorded and that the information gathered would be kept confidential.

The duration of the interviews ranged from 30 minutes to one hour. All the interviews were carried out in English and transcribed verbatim. A few Arabic terms were used by some of the Omani participants, and AA translated these into English.

AA discussed the findings of the first 3 interviews with the research team separately after each interview. The first 3 interviews then contributed to the development of the interview guide (see additional file 1). Subsequent questions and probes were also modified, based on the findings of these 3 interviews. The last 14 interviews were then conducted. The transcriptions where made immediately after each interview.

The interviews focused on the perceived challenges faced by health care providers in the study context with regard to TB. The interviews also explored the roles played by the private sector and the expatriate population in relation to TB control in the country. The questions were semi-structured and probing was used when appropriate.

### Analysis

The interviews were analyzed using content analysis [10]. AA read through the transcripts several times to get fully immersed in the material codes, and categorizations were then created inductively in steps. The meaning units were first identified by highlighting phrases in the transcripts and were then labeled as condensed meaning units (CMU). The CMUs were labeled with codes, and different sub-categories were created, by grouping together similar codes. Different comparable sub-categories were then assembled to make Categories. Categories were analyzed and combined into 3 different themes. Table 1 gives an example of the analysis process. The coding and analysis were carried out by AA, and then together with the second author (ADC). Categories, sub themes and themes were arrived at by consensus between the four authors.

**Table 1:** An example of the analysis process

Theme 1	Underprivileged expatriates: The threat of repatriation		
**Category**	**TB- an imported foreign disease**	**Living on the fringe of the society**	**Counter-productive repatriation policy**

Codes	Bring infection with them	Poor	Unacceptable
	Come from high incidence	Living in crowds	Not ethical
	Need Strict entry	No health insurance	Needs review
	Need Stringent screening	Poor nutrition	Repatriation Fear
		Treated poorly by sponsors	Hide
			Escape
			Travel for treatment

### Ethical considerations

Participants were informed about the voluntary nature of the study and confidentiality of the data and were given choice not to participate in the study. The study was approved by the medical research and ethics committee (MREC), Sultan Qaboos University, College of Medicine and Health Sciences, Muscat, Oman.

## Results

Three themes emerge from the content analysis. The first explores the threat of repatriation faced by underprivileged expatriates. The second highlights the private sector as a criticized and forgotten key player in the control of TB. The third describes the user and provider barriers faced by Omani patients in the Omani public health system.

### Theme 1: Underprivileged expatriates: The threat of repatriation

This theme emerged from health care providers perceptions of how expatriates impact on the TB burden and on TB control in Oman. They perceive TB as an imported disease, which is brought into Oman by expatriates. However, they also recognized that expatriates live on the fringes of Omani society and are disadvantaged socially and economically by the existing policy of repatriation.

#### TB- an imported foreign disease

Some of the participants tended to indict expatriates as infection importers who bring TB into Oman. They believe that the increasing number of expatriates arriving in Oman from high incidence countries have prevented Oman from achieving it's TB elimination targets. Most of the public health system participants also suggested the need for strict control of expatriates' entry into the country, especially in relation to those coming from high prevalence regions. They also considered that the current screening tests for expatriates were insufficient and sometimes ineffective. They suggested more stringent screening tests of expatriates before and after their arrival into the country.

"I think expatriates are the most important cause of the prevalence of TB in Oman, especially expatriates coming from Asia ... it may take three months for him to come for the check-up. So during this period he can infect persons from Oman... I think especially for TB, it is not enough to screen them every 2 years; they should be screened every 4 or 6 months." (Public sector, expatriate)

#### Living on the fringes of the society

Both public and private participants described the expatriates, mainly labor workers, as socially and economically vulnerable. Most of the public health providers we interviewed acknowledged that there was no information available on the health status of these workers, as they mostly go to private practitioners. Furthermore, most participants highlighted how such workers frequently have poor living conditions, and may be unable to pay for health care. The institution of compulsory health insurance for these workers was suggested as a means of insuring that expatriates seek health care.

"The living conditions, hygiene and crowdedness are causes of spreading among expatriates. If you take the upper class of the expatriates they rarely have TB, it is the lower class that have it. We have to maintain some basic standards of life for them as a policy" (Private, expatriate).

#### Counter-productive repatriation policy

The participants pointed to the social and economic disturbances imposed on the expatriates diagnosed with TB, which results from the repatriation policy. In addition, all participants said that this policy might cause expatriates to hide and travel home before treatment. They urged that this policy be re-evaluated, and that policies aimed at improving the health seeking behaviors of the expatriates, be adopted.

"We had a staff nurse who had small cavitations in her lungs. She told me: 'what do you think I have'. I told her that being from India, TB should be kept in mind. Next day the patient is not on the bed. She went home on leave ....we should treat expatriates like Omanis. Deporting them is against human rights" (Public, Omani)

### Theme 2: The private sector: a criticized and forgotten key player

This theme emerged from both public and private providers' perceptions of the private health care sector's role in TB care and control in Oman. Most of the public health sector participants addressed the need for better regulation of the private clinics and hospitals by the Ministry of Health (MOH). The public health care providers believe that private health practitioners are primarily driven by their clients' demands rather than by best practice management principles. The participants have also pointed to existing suboptimal competencies and poor infrastructure in private practices.

#### The need for better regulation and involvement of the private health sector

The participants in the public health sector perceived a necessity for better regulation of private health care practices, by the responsible bodies at MOH. They also stated the need for tighter control of the private sector, through increased supervision, and suggested linking practice licenses to knowledge of and adherence to MOH policies. On the other hand, all private participants expressed the view that the private sector needed to be more involved with the National TB Control Program (NTBCP); in particular through improved communications, and through the referral system between the private clinics and the NTBCP. Private participants also acknowledged the need for formal inter-sectoral cooperation.

"There should be some communication with MOH regarding the infectious diseases especially TB, what to do when you come across TB cases, where to send? There it should be regular communications. (Private, expatriate)

#### Private practitioners influenced by commercial incentives

Most of the public sector participants criticized the private practitioners for being influenced by the pursuit of financial gain in their practices, which could have the effect of compromising medical care in this sector. This was seen as having negative consequences on medical practice in Oman. They also tended to describe private sector practitioners as primarily driven by client demands, rather than by disease control and management requirements. Some public health participants stated that private practitioners might deliberately avoid reporting expatriate TB patients, and advise them to go for treatment in their home countries, to help them avoid repatriation.

"Whenever an expatriate patient has cough he will go to private (doctor) and even if the doctor suspected TB he will ask him to hide himself. "Don't go to government hospital, they will catch you and send you back home" And he will ask him to take leave and go back to his country "take your treatment and come back". (Public, Omani)

The private providers, on the other hand, said that they had to ensure their practices remained competitive with those of other private providers. Financial profit was thus seen as an important and inevitable driving force in their daily work.

"The health centers take a longer time in waiting and watching and to (carry out) investigations (examinations). As for the private (practitioners), they are here to survive and to make money. If a patient wants to have an investigation which private clinic will not do it?!!". (Private, expatriate)

#### Suboptimal competencies and poor infrastructure in the private sector

Most of the public health providers also described private practitioners as having poor knowledge and competency in general, and specifically in relation to TB care. The private sector was also criticized for its insufficient and ineffective contribution to TB care and control. Meanwhile, the private practitioners attributed their difficulties in TB care to the lack of proper facilities and training in relation to TB diagnosis and treatment.

"The private needs to look at the quality in their labs. I'm sure that there are cases that are being missed. They have small labs. The stains are there for years in those labs". (Private, Omani).

### Theme 3: Omani patients and the public health system: user and provider barriers

This theme emerged from categories concerning the participants' experiences and perceptions of Omani patients seen in the public health system. The first category was related to socioeconomic access barriers experienced by Omani TB patients. Patients are perceived as poor, ignorant and stigmatized. The second category focused on the need to strengthen human and material capacity, as well as to improve policies in the public health system.

#### Socioeconomic access barriers to TB care

Participants reflected on their experiences and perceptions of Omani patients seen in the public health system. The public sector participants referred to their patients as malnourished and living in poor, crowded conditions. They linked this to poverty and low income. In addition, the health care providers said that patients' ignorance of TB as a disease, and the risk of being stigmatized, contributed to difficulties in getting proper care.

"I feel that poor housing is playing a role in this ... I see that patients are in poor housing and they don't have good nutrition. For example out of twenty patients, fifteen of them are those who are earning for their families, and they have low income and poor job, and every day life expenses are increasing" (Public, Omani)

#### The need to strengthen capacity and improve polices in the public health system

All the public health sector participants interviewed, acknowledged the activities of the TB control program, and were aware of how the TB notification and surveillance system functioned. However they believed that there were insufficient facilities dedicated to TB care, including health care providers, clinics and hospitals.

They also perceived that the low quality of the existing TB facilities along with inadequate safety measures presented obstacles to TB control. Some of the private sector participants described the public health system as being 'academic', and characterized by long waiting times. Furthermore, most of the public sector participants pointed out that GPs had insufficient knowledge about TB and did not know when to suspect it's presence. They suggested that this has resulted in TB cases being missed at the level of primary care, which in turn led to delays in diagnosis.

"I realized people have forgotten about TB. The physicians, the general practitioners they think that TB doesn't exist in Oman, even though there are guidelines. (Public, Omani)

Though all the public health participants appreciated the decline in TB over the last three decades, some expressed the need to change current guidelines and policies. They felt that some polices were obstacles to TB control. Some of the current policies were criticized, e.g. admission for two months of smear-positive patients. In addition, health care providers perceived Oman as a low incidence country, and recognized the need to adopt policies of low incidence countries.

" Policy makers are sticking very strictly to the WHO recommendation of TB management for third world countries and those countries are high incidence (ones) ... we are more like European countries, so we should follow their policies". (Public, Omani)

## Discussion

In many developed countries with a low incidence of TB, the number of migrants from high incidence countries has been increasing [9]. As a consequence, TB associated migration has become an important public health issue in low incidence countries [8]. Many of these countries have legislation in place with regard to temporary migrants, such as visitors, foreign students and skilled and unskilled labour. Although the expected individual and public health benefits of these legislations have been questioned [11], legal migrants are either screened offshore before departure, e.g immigrants to US or Canada, or are screened on arrival, as in Europe [8].

In Oman, migrants (expatriates) are mainly temporary labour workers from the Indian subcontinent. They are screened for active TB in their home country before being granted their working visa permit. Repeat screening is carried out within one month of arrival, and then every 2 years. If active TB cases are confirmed, or individuals are suspected of having TB, based on abnormal chest x-rays, they are normally not granted a visa. Furthermore, as an extra measure to prevent TB transmission, expatriates developing TB during their stay in the country, are deported after conversion to smear negative. This deportation is commonly referred to as "the repatriation policy". This has been a major issue of discussion during the interviews. The overall perception was that repatriation of expatriates might present a major challenge to TB care and control in Oman. It is known from research in other countries, that fear of repatriation prevents expatriates from accessing health care services, especially when they know that they have TB [12]. Similar concerns have also been expressed by many of the participants in our study. Examples of expatriates with TB going unreported were given, based on participant's experiences. Therefore, the repatriation policy is seen as imposing a barrier to early detection and effective treatment of expatriates, which in turn affects overall TB control in the country. To improve TB control among expatriates in Oman, we suggest that this policy be revised, and that alternative policies and strategies be developed.

These could include short-term interventions, focusing on early detection and treatment of active TB and latent TB, among expatriates, rather than on post treatment deportation. Another strategy might be to increase efforts towards global control of TB, especially in high incidence countries. These could include technical and financial support for TB control programs, as well as funding for further research, which could result in more cost-effective and sustainable outcomes[13].

In Oman, health care providers perceive the majority of expatriates' as living on the fringes of society and as having many socioeconomic difficulties. Expatriates are therefore more likely to fall outside the overall health care system, where their access to and utilization of the health care system in Oman is largely unknown. National disease control strategies, not merely TB control strategies, should give more attention to optimizing the socioeconomic environment of expatriates and to improving access to affordable and appropriate health care services. To address the issues faced by underprivileged expatriates we suggest interventions to improve housing plus overall living circumstances, and to institute a health insurance system for expatriates.

Expatriates in Oman are mainly served by the private health care system, because the majority of them work in the private sector [3]. There are few statistics relating to the health care services provided by the private sector in many countries, including Oman [14]. Overall, the private sector is overlooked and not integrated into TB control planning. Due to economic development, and in response to an increasing number of expatriates, the number of private practices has increased significantly over the last 10 years in Oman [1]. In our study, participants said there was a lack of integration and collaboration between the private and public health systems. They said that the private health care system was being overlooked, as it is not directly involved in TB control. The failure to integrate the private health care system into the overall national health care system acts as an impediment to disease control strategies, especially when it comes to TB [15]. For TB care delivery to improve, contextualized strategies and policies, to regulate and integrate the private sector are needed. However, involving the private health care system in TB care is not yet simple and straightforward. Public practitioners have expressed concerns about the priorities of the private sector and perceive private practitioners as being overly influenced by economic imperatives. In addition, private practitioners have accused the public health practitioners of being inefficient in the care process. This conflict of values between the two sectors makes it difficult to have a Private Public Mix (PPM) strategy, without an in-depth analysis of the factors operating in the relationship between the two sectors [16].

The findings of the study also uncovered some hidden problems relating to private health care delivery in the country. The participants, including some of the private health care providers themselves, questioned the effectiveness of the private health care system. This leads to the conclusion that it will be necessary to evaluate the performance and competencies of the private care system and the services it provides. A previous study has shown that there is a significant difference between private and public practitioners in Oman when it comes to diagnosis and knowledge of TB and that it is the private practitioners who have less knowledge and ability to diagnose than the public ones [17].

Moreover TB, more than other diseases, is known to expose weaknesses in health systems [18]. The study has also revealed the existence of public health system barriers that are pervasive throughout the present national TB control program. Shortage of resources, both human and material, as well as inadequate case detection process due to a low rate of diagnosis and knowledge of TB, are the most prominent health system barriers perceived by the participants. Such health system barriers suggest the need for structural performance evaluation of the quality of TB care at primary, secondary and tertiary levels of the public health system. The report on Country Cooperation Strategy for WHO and Oman 2005-2009, has acknowledged the need to strengthen the health system in Oman by increasing health financing and institutional capacity and improving service delivery [19].

The study has also found that health care providers perceived Omani TB patients as being poor, ignorant about their health and stigmatized by TB. TB is well known as the disease of the socioeconomically disadvantaged [20] with more than 90% of the Omani TB patients earning less than the gross national income per capita[6]. Health system barriers to already vulnerable groups of individuals may adversely influence TB control in the country. Therefore, curtailing TB incidence among nationals, to achieve elimination targets, entails vigilant interventions at both patient and health system levels simultaneously. Educating TB patients, and improving their socioeconomic status is not enough, unless health system related barriers are removed through enriched TB control resources and improved policies.

### Methodological considerations

In this study, we explored the perceptions, as well as the experiences of different health care providers, working in a number of settings. The selection of different health care providers from both public and private health sectors served to triangulate the data that emerged in the study. However, It should be noted that the patients' perspective was not taken into account, and we acknowledge the need to explore the patient's perceptions and experiences, so as to capture the full picture of the barriers to better TB care and control in the country.

The interviews were carried out in English, which is not the mother tongue of many of the health care providers. However, Omani participants were encouraged to use Arabic terms, whenever they found it difficult to use the English words or expressions. The interviewer works in a public university, and this may have made the private participants more hesitant in expressing all their perceptions. Similarly, the public participants may have been reluctant to express strong views regarding the public health system, as it is their employer. However, the findings do reveal several critical views of the public health care sector, which suggests that the interview situation did allow for discussion of sensitive topics. Also, as the interviewer is familiar with the Omani health system, he was able to contextualize the participants' accounts and to ask relevant follow-up questions.

Transferability is the term used in qualitative research in parallel to the external validity or generalizability in quantitative research [21]. The transferability of the results of this study must be theoretical or analytical since the interviewees are purposively selected [22]. This means that we can generalize the results to contexts that theoretically are similar to the one studied. However, there are only 17 purposively selected interviewees from Muscat governorate; these may not be representative of the entire country context. Thus, this may limit the transferability of the results.

## Conclusion

This study, a first of its kind in Oman, has identified some of the challenges and barriers to TB control in the country based on health care provider's perceptions and experiences. These challenges mainly relate to unintended negative consequences arising from the current repatriation policy of immigrants and to and the lack of involvement of the private sector in TB control. Therefore, TB control strategies designed to address these challenges are needed, for Oman to reach it's TB elimination targets.

## Competing interests

The authors declare that they have no competing interests.

## Authors' contributions

AA was involved in study design, data collection, data analysis and Manuscript writing. AD was involved in study design, data analysis and manuscript writing. AO was involved in study design and manuscript writing. GF was involved in study design and manuscript writing. All authors read and approved the final manuscript.

## Authors Information

The researchers are a) an Omani epidemiologist with experience in public health and TB epidemiology in Oman, b) an Omani associate professor in pulmonary medicine, c) an Indian researcher who has worked on qualitative research in India and d) a Swedish researcher who has carried out qualitative research pertinent to TB in Asia. Ethical approval was obtained from the Research and Ethics committee and the College of Medicine and Health Sciences at Sultan Qaboos University, Oman.

## Pre-publication history

The pre-publication history for this paper can be accessed here:

http://www.biomedcentral.com/1472-6963/10/210/prepub

## Supplementary Material

Additional file 1 **The interview guide**. This file contains the interview guide used to collect the data from the participants in this study. Click here for file
