# Supplementary material for: Immigrants and health system challenges to TB control in Oman
Source: BMC Health Serv Res. 2010 Jul 16;10:210. doi: 10.1186/1472-6963-10-210 (PMC2919530; doi:10.1186/1472-6963-10-210)
Supplement: Additional file 1 — The interview guide. This file contains the interview guide used to collect the data from the participants in this study. [file 1472-6963-10-210-S1.DOC]

**Interview Guide**

1. Introduction:

How long have you been working with TB patients?

How often do you see TB patients?

2. Could you please tell me about your experiences when seeing a TB patient in your setting? Let’s start by describing your good experiences when seeing TB patients?

Follow-up:

- Do you have difficulties in your clinic/hospital when managing TB patients?
- Do you have difficulties related to the patient themselves that affect their diagnosis and management?

3. In general, what do you think about TB control in Oman?

Follow up:

- What do you think about the TB incidence/prevalence in Oman?
- Would you please tell me what do you think about DOTS strategy for TB control in Oman?
- What do you think about guest workers and TB control in Oman?
- Could you tell me about what do you think about private sector to TB control in Oman?
- From your point view, what are the difficulties that are facing better TB control in Oman?

4. In general, would you please tell me your view in how Oman would have a better TB control?

Follow-up:

Is it possible to eliminate TB? If yes How if no why?

Do you have some other recommendations for the TB control program in Oman?
